# Dengue in deployed military personnel, 1905–2024: a systematic review of incidence, diagnostics and prevention

**DOI:** 10.1093/jtm/taaf120

**Published:** 2025-11-24

**Authors:** Eric Agboli, Hanna Jöst, Dimitrios Frangoulidis, Le Huu Song, Do Duc Anh, Antonios Katsounas, Thirumalaisamy P Velavan, Jonas Schmidt-Chanasit

**Affiliations:** Department of Arbovirology and Entomology, Bernhard Nocht Institute for Tropical Medicine, 20359 Hamburg, Germany; Department of Biomedical Sciences, University of Health and Allied Sciences, Ho PMB 31, Ghana; Department of Arbovirology and Entomology, Bernhard Nocht Institute for Tropical Medicine, 20359 Hamburg, Germany; H/MI2 Department, Bundeswehr Medical Academy, 80937 Munich, Germany; Vietnamese German Center for Medical Research, Hanoi 100000, Vietnam; 108 Military Central Hospital, Hanoi 100000, Vietnam; Institute of Tropical Medicine, University of Tübingen, 72074 Tübingen, Germany; Vietnamese German Center for Medical Research, Hanoi 100000, Vietnam; Institute of Tropical Medicine, University of Tübingen, 72074 Tübingen, Germany; Faculty of Medicine, Ruhr University Bochum, 44801 Bochum, Germany; Department of Medicine, Infectious Diseases and Critical Care Division, Knappschaft Kliniken University Hospital Bochum, 44892 Bochum, Germany; Institute of Tropical Medicine, University of Tübingen, 72074 Tübingen, Germany; Faculty of Medicine, Duy Tan University, Da Nang 550000, Vietnam; Department of Arbovirology and Entomology, Bernhard Nocht Institute for Tropical Medicine, 20359 Hamburg, Germany

**Keywords:** Dengue, military deployments, endemic regions, diagnostics, prevention, force health protection

## Abstract

**Background:**

Military deployments to dengue-endemic regions present ongoing risks to health and mission readiness. This review synthesizes a century of evidence on the incidence, clinical features, diagnostics and prevention of dengue in military personnel, aiming to guide future health policies, research and Force Health Protection strategies.

**Methods:**

Using Preferred Reporting Items for Systematic Reviews and Meta-Analyses (PRISMA) 2020 guidelines, a search of PubMed and Google Scholar (March 15–April 5, 2025) identified 32 English-language studies (1905–2024) reporting primary data on dengue in military personnel. Studies were selected based on predefined criteria and narratively synthesized.

**Results:**

A review of 32 studies involving 42 272 military personnel across 41 deployment settings identified 69 224 dengue cases, with outbreaks dating back to 1904. A notable spike occurred between 2012 and 2017, likely due to increased deployments to endemic regions and better surveillance. Diagnostic methods have advanced from early clinical recognition to modern Non-structural Protein 1 (NS1) antigen and Polymerase Chain Reaction (PCR) tests. Common symptoms included high fever, intense headache and myalgia. Despite efforts such as integrated vector control and Personal Protective Measures (PPMs), and new vaccines (Qdenga®, Takeda), prevention remains limited by inconsistent use of integrated vector control and PPMs, low vaccine uptake and eligibility constraints.

**Conclusion:**

Dengue continues to threaten operational readiness in tropical deployments. Strengthening integrated vector control, PPMs, vaccination and real-time surveillance is crucial to reduce its impact and control other co-endemic diseases like malaria, yellow fever, chikungunya and Zika. Future research should focus on evaluating integrated vaccine and vector control strategies aimed at enhancing Force Health Protection among military personnel.

## Background

Dengue is a viral illness spread by mosquitoes, specifically *Aedes aegypti* and *Aedes albopictus,* and is caused by four distinct serotypes of dengue virus (DENV-1 to DENV-4).^1^ Globally, 136 countries or territories have reported current or prior autochthonous dengue transmission. In 2024, over 14.2 million dengue cases were reported by WHO, including 7.5 million confirmed cases, over 52 000 severe cases and more than 10 000 deaths.^1^ Clinically, dengue can present as a mild fever but may also progress to severe life-threatening conditions such as dengue hemorrhagic fever (DHF) and dengue shock syndrome (DSS).^2^^,^^3^ In recent decades, the incidence of dengue has increased globally, driven by factors like rapid urbanization, shifts in climate, rising populations and increased international mobility.^1^^,^^4^^,^^5^ This expanding burden poses challenges not only for civilians but also for military personnel deployed in areas, where dengue is common.

Military personnel face heightened risks of dengue infection due to deployments in tropical and subtropical regions where the virus is endemic. Extended outdoor activities, frequent movement and limited access to medical care increase exposure risk during such missions. Historical records indicate that dengue has affected military forces for decades, though documentation is often incomplete. As early as World War II, Sabin’s 1952 report described dengue cases among soldiers.^6^ Later, during peacekeeping missions in the late 20th century—particularly Operation Restore Hope in Somalia (1992–1993)—US troops experienced confirmed dengue infections, marking one of the earliest recognized outbreaks involving military personnel in Africa.^7^^,^^8^ Research on US military operations in the early 1990s estimated an infection incidence of 1.5%, equivalent to 17.6 new seroconversions per 10 000 deployment-months.^9^ Subsequent outbreaks further highlighted the operational impact of dengue: in 1984, 24 American service members in the Philippines were hospitalized with dengue symptoms, resulting in an average of six hospital days and nine duty days lost per person^10^; and in 2013, a large-scale outbreak at a South Indian military base led to the hospitalization of 266 soldiers within two months.^11^ Collectively, these events illustrate the persistent threat dengue poses to deployed forces and underscore the need for improved surveillance, preventive measures and recordkeeping in military medical research.

Diagnosing dengue in field settings remains challenging due to limited laboratory infrastructure, despite advancements in portable PCR platforms and combined antigen–antibody rapid tests that enhance detection with greater accuracy.^12^^,^^13^ A report highlighted the need for active surveillance and timely diagnosis.^14^ However, ensuring early, reliable detection across diverse deployment environments still depends on accessible, user-friendly diagnostic tools requiring minimal technical expertise.^15^

Dengue can be controlled and prevented in the military. Integrated vector control is a core preventive strategy. A study showed that camps using targeted vector control interventions had markedly lower infection rates.^16^ The US military has advanced prevention through permethrin-treated uniforms; N, N-diethyl-meta-toluamide (DEET) repellents; treated bed nets; and insecticide fogging,^17^ while also contributing to dengue vaccine development.^18^ Although newer vaccines show promise for protecting deployed personnel,^19^^,^^20^ challenges remain, including inconsistent implementation of integrated vector control strategies, variable compliance with protective measures, limited surveillance and low adherence to preventive behaviours.^21^ These issues highlight the need for sustained education and behaviour-change programs to ensure effective protection in the field.^22^

Until now, existing data on dengue in the military context has been fragmented. A systematic review is needed to consolidate knowledge, identify trends and evaluate interventions’ effectiveness. The findings aim to inform strategies, strengthen pre-deployment protocols and support integrated military-civilian approaches to dengue prevention and control.

## Materials and methods

### Search strategy and data sources

We conducted a systematic review of peer-reviewed literature to identify studies reporting on dengue virus infections among military personnel. The literature search was performed between March 15 and April 5, 2025, using the databases: PubMed and Google Scholar. These databases were selected to ensure broad coverage of biomedical and interdisciplinary research. PubMed provided comprehensive indexing of clinical and epidemiological studies, while Google Scholar captured relevant grey literature and institutional publications. Search terms included various combinations of the following keywords: ‘dengue in the military,’ ‘dengue in the military context,’ ‘dengue in the armed forces,’ and ‘dengue in the armed forces context.’ Boolean operators were used to optimize the search strategy (e.g. dengue AND military OR dengue AND armed forces). ‘How common is dengue among soldiers?’, ‘Incidence of dengue in armed forces deployments’, etc.).

To ensure relevance and methodological quality, studies were included in the review if they met predefined eligibility criteria. Eligible studies were required to be published in journals in English and to report original data on dengue infections in military populations. This included studies addressing incidence rates, outbreak investigations, diagnostic approaches, or prevention strategies. Additionally, studies were required to provide specific contextual details, such as the geographic location, time period and characteristics of the military personnel involved. Exclusion criteria were also clearly defined. We excluded articles not in English, editorials, narrative reviews, conference abstracts without accessible full text and studies focusing exclusively on civilian populations.

A recent publication about arboviruses in the UK Armed Forces^23^ was excluded from the current study. This is because the recent publication was not within our search timeline (as shown in our search criterion). The data was extracted from electronic health records and statutory notifications, which were not available online at the time of our search. The study also has a selection bias, as it includes only laboratory-confirmed severe dengue cases, and some cases occurred outside a military context. Notwithstanding, the authors advised that data from this recent publication is an addition to our current study, showing a worldwide overview of dengue in the military context.

### Screening and data extraction

All records identified through database searches were imported into EndNote X9 (Clarivate Analytics) for reference management. Duplicate entries were removed using EndNote’s built-in de-duplication function, followed by manual verification to ensure accuracy. Two independent reviewers screened titles and abstracts to assess initial relevance, obtaining full-text articles for all studies that were potentially eligible. Full-text articles were then reviewed in detail against the inclusion criteria. Discrepancies in study selection were resolved through discussion and, when necessary, adjudication by a third reviewer.

**Figure 1:**
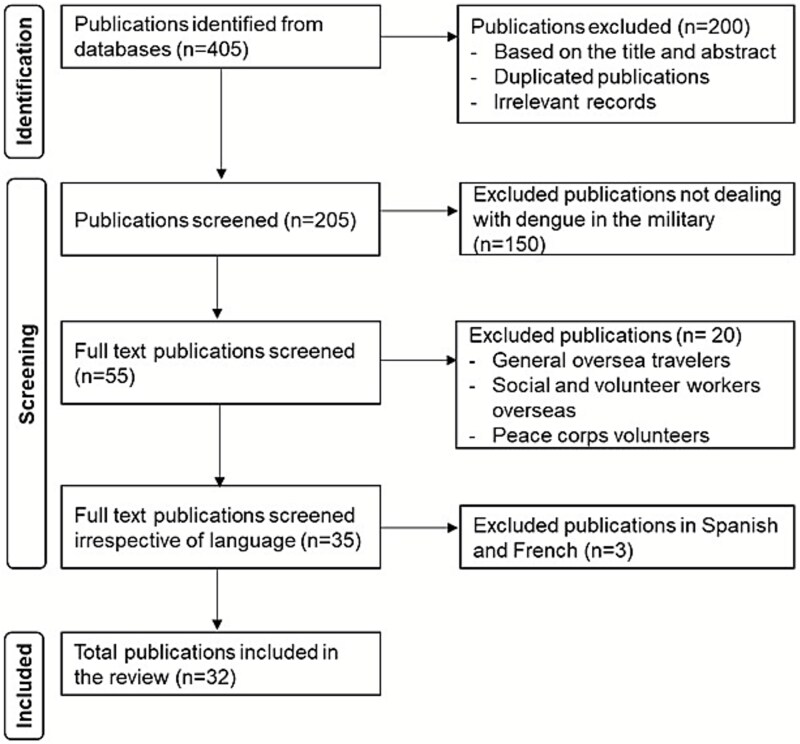
PRISMA flow diagram depicting the screening and inclusion process of studies on dengue infections in military populations.

Data extraction was conducted using a structured Excel template developed prior to the review. For each included study, the following information was systematically recorded: year of publication, country deploying military personnel, country and/or region of dengue detection, study design, sample size, number of reported dengue cases and diagnostic methods employed. Key findings and study limitations were also noted. Two authors extracted data independently and cross-checked entries to ensure consistency and accuracy. The review was conducted in accordance with the PRISMA 2020 guidelines.^24^ A PRISMA flow diagram (Figure 1) summarizes the study selection process, detailing the number of records identified, screened, excluded and included in the final synthesis.

## Results

**Figure 2:**
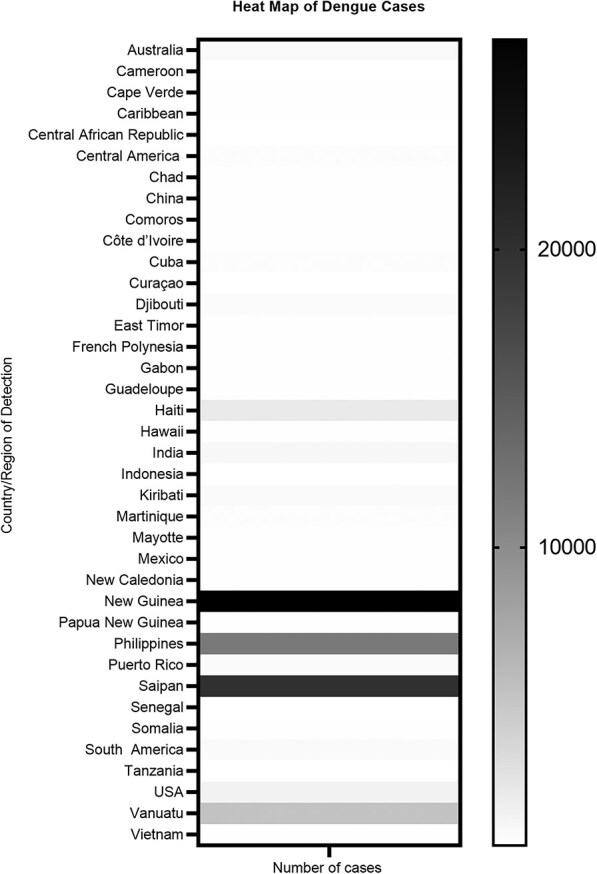
Heat map of geographic distribution of dengue detection studies by country or region (1905–2024).

**Figure 3:**
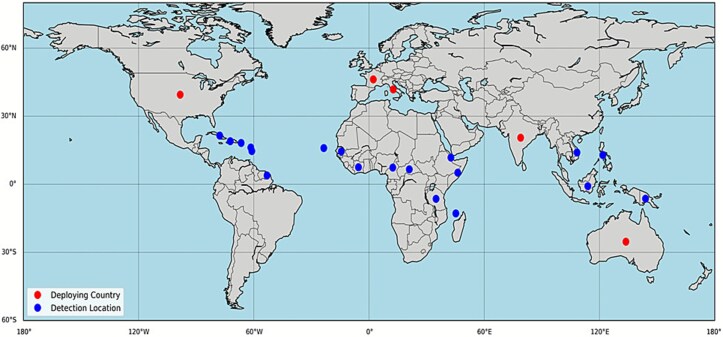
Global map of dengue detection and military deployment locations. Map generated using Python (Matplotlib v3.8, Basemap Toolkit), based on compiled surveillance and deployment data from reviewed literature. The map illustrates countries that deployed military personnel (red dots) and locations where dengue was detected (blue dots). This figure was generated based on a compilation of publicly available data extracted from published studies listed in Table 1 of this review. Geographic coordinates were assigned using approximate country centroids, and the map was visualized using Python with the Matplotlib and Basemap libraries.

A total of 405 records were identified through database searches. Following the removal of duplicates and screening based on inclusion criteria, 32 studies were selected for the final review (Figure 1). The 32 studies involving 42 272 military personnel across 41 global deployment locations identified 69 224 cases of dengue with all the four serotypes (Table 1). The high number of cases compared with the total military personnel was due to missing information, such as sample size, in some of the studies. The selected studies spanned the publication years 1905 through 2024. Over this period, reporting on dengue in military contexts increased, peaking between 2012 and 2017 in terms of study output and reported cases. This surge may be related to the frequency and nature of deployments in that decade, as many operations took place in dengue-endemic areas alongside improved disease surveillance. Most included studies were retrospective, while three employed a case study, and nine were prospective (Table 1). USA and France were the most common troop-deploying nations, with most deployments across Africa and Asia (Table 1, Figures 2 and 3).

**Table 1:** Summary of reported dengue cases in military deployments by year, deployment country, and detection region (1905–2024)

Reporting year	Armed forces of country	Country of detection	Study design	Sample size (n)	Number of reported cases (n)	Detection method	Reference
2024	India	India	Prospective	422	62	IgG, IgM	^28^
2023	USA	USA	Retrospective	1606	971	NS1, Serology (IgM and IgG), PCR	^25^
2022	USA	Puerto Rico	Cross-sectional	494	330	FlowNT, PRNT, Serum Dilution Testing	^26^
2022	Australia	Papua New Guinea	Seroprevalence	208	19	NT	^27^
2021	USA	Djibouti, Philippines	Retrospective	1068	60	Serology, PCR, Immunohistochemistry	^29^
2017	USA	Americas (Central/South), Asia, Africa	Retrospective	1000	76	NT	^9^
2017	USA	America (Central/South), Mexico, Caribbean	Prospective/Observational	108	5	ELISA, PRNT, PCR	^30^
2015	USA	Curaçao	Case study	6	1	IgG	^31^
2014	India	India	Observational retrospective	266	192	NS1 antigen ELISA	^11^
2014	USA	America (Central/South), Asia, Africa	Serosurveillance	414	55	Microneutralization assay	^32^
2013	France	Haiti	Surveillance	97	4	ELISA, IgG, PCR, Cell culture, NS1	^32^
2013	France	New Caledonia	Surveillance	3090	2	ELISA, IgG, PCR, Cell culture, NS1	^32^
2013	France	French Polynesia	Surveillance	2467	2	ELISA, IgG, PCR, Cell culture, NS1	^32^
2013	France	Tanzania	Surveillance	n/d	1	ELISA, IgG, PCR, Cell culture, NS1	^32^
2013	France	Mayotte	Surveillance	823	2	ELISA, IgG, PCR, Cell culture, NS1	^32^
2013	France	Côte d’Ivoire	Surveillance	972	1	ELISA, IgG, PCR, Cell culture, NS1	^32^
2013	France	Djibouti	Surveillance	2882	4	ELISA, IgG, PCR, Cell culture, NS1	^32^
2013	France	Martinique	Surveillance	2384	164	ELISA, IgG, PCR, Cell culture, NS1	^32^
2013	France	Indonesia	Surveillance	n/d	1	ELISA, IgG, PCR, Cell culture, NS1	^32^
2013	France	Guadeloupe	Surveillance	1747	100	ELISA, IgG, PCR, Cell culture, NS1	^32^
2013	France	French Guiana	Surveillance	4012	49	ELISA, IgG, PCR, Cell culture, NS1	^32^

(*Continued*)

**Table 1:** Continued

Reporting Year	Armed forces of Country	Country of detection	Study design	Sample size (n)	Number of reported cases (n)	Detection method	Reference
2012	France	Cameroon	Surveillance	2423^*^	1	PCR	^33^
2012	France	Cape Verde	Surveillance	2423^*^	5	Cell Culture	^33^
2012	France	Central African Republic	Surveillance	2423^*^	1	Serology	^33^
2012	France	Chad	Surveillance	2423^*^	28	Serology	^33^
2012	France	Comoros	Surveillance	2423^*^	3	PCR, Cell culture	^33^
2012	France	Côte d’Ivoire	Surveillance	2423^*^	13	PCR, Cell culture, Serology	^33^
2012	France	Djibouti	Surveillance	2423^*^	164	PCR, Cell culture, Serology	^33^
2012	France	Gabon	Surveillance	2423^*^	23	PCR, Serology	^33^
2012	France	Mayotte	Surveillance	2423^*^	1	Cell culture	^33^
2012	France	Senegal	Surveillance	2423^*^	1	PCR	^33^
2012	France	Somalia	Surveillance	2423^*^	1	Cell culture	^33^
2012	USA	New Guinea	Review	n/d	2960	n/d	^34^
2012	USA	New Guinea	Review	n/d	24 079	n/d	^34^
2012	USA	Philippines	Review	n/d	8926	n/d	^34^
2012	USA	Philippines	Review	n/d	2012	n/d	^34^
2012	USA	China	Review	n/d	40	n/d	^34^
2012	USA	Kiribati	Review	n/d	396	n/d	^34^
2012	USA	Saipan	Review	n/d	20 000	n/d	^34^
2012	USA	Vanuatu	Review	n/d	5000	n/d	^34^
2012	USA	New Caledonia	Review	n/d	n/d	n/d	^34^
2012	USA	Hawaii	Review	n/d	56	n/d	^34^
2012	USA	Australia	Review	n/d	n/d	n/d	^34^
2008	USA	Philippines	Case study	1	1	IgM, PCR	^35^
2008	France	French Guiana	Retrospective	3000	149	PCR, ELISA (IgM)	^36^
2006	USA	South America	Case study	1	1	IgM	^37^
2004	USA	Philippines	n/d	n/d	800	n/d	^38^
2003	Italy	East Timor	Cross-sectional	595	16	NT, HI, PRNT	^39^
2002	Australia	East Timor	Prospective	2500	9	IgM	^40^
1999	Multinational	Haiti	Retrospective	7200	1600	Serology (IgM)	^41^
1999	Multinational	Haiti	Retrospective	249	41	ELISA (IgG, IgM)	^8^
1997	USA	Haiti	Serosurveillance	22	8	IgM	^14^
1995	USA	Somalia	Prospective	96	41	Cell culture	^7^
1994	USA	Haiti	Prospective	106	24	Serology (IgM)	^42^
1994	USA	Somalia	Prospective	90	15	IgM, IFA, PCR, Cell culture	^43^
1989	USA	Philippines	Retrospective	n/d	24	HI, Cell culture	^10^
1969	USA	Vietnam	Prospective	94	10	HI	^44^
1969	USA	Vietnam	Prospective	103	9	HI, PRNT	^45^
1969	USA	Vietnam	Retrospective	1616	15	HI	^46^
1967	USA	Vietnam	Prospective	110	31	HI	^47^
1945	USA	Vanuatu	n/d	n/d	n/d	n/d	^48^
1944	USA	Australia	n/d	n/d	100	n/d	^49^
1912	India	India	n/d	n/d	319	n/d	^50^
1905	USA	Cuba	n/d	n/d	200	n/d	^51^

n/d = no data; ^*^ = total deployments to 11 different countries with individual case numbers; PCR = polymerase chain reaction; ELISA = enzyme-linked immunosorbent assay; PRNT = plaque reduction neutralization test; FlowNT = flow cytometry-based neutralization test; IFA = immunofluorescent assay; HI = hemagglutination-inhibition; NT = neutralization test; NS1 = non-structural protein 1.

Recent surveillance efforts (2022–2024) underscore renewed attention to dengue in military settings, with studies from India, Australia and the USA involving sample sizes ranging from 208 to over 1000 individuals.^25–27^ One large retrospective USA military study reported 971 dengue cases among 1606 personnel, using NS1 antigen detection, serology and PCR.^25^ In 2024, a prospective Indian study identified via Immunoglobulin G (IgG) and Immunoglobulin M (IgM) testing 62 cases among 422 individuals over one transmission season (an incidence of 147.4 per 1000 over 6 months).^28^ Similarly, a 2015 cross-sectional US serosurveillance in Puerto Rico employed advanced methods, Flow Cytometry-based Neutralization Test (FlowNT) and Plaque Reduction Neutralization Test (PRNT) to detect evidence of dengue infection in 330 out of 494 troops (66.8%), underlining the high baseline exposure in endemic areas.^26^

Earlier research provides evidence of persistent dengue risk across multiple decades and deployment sites. Reported outbreaks varied widely in magnitude and context. A significant cluster of studies in 2010-2011 conducted by French military surveillance teams reported dengue activity in more than 15 locations, including Martinique, Guadeloupe, French Guiana, Djibouti and the Pacific territories.^32^ These studies often employed a standardized diagnostic panel combining Enzyme-Linked Immunosorbent Assay (ELISA), PCR, NS1 antigen testing, and virus culture. On the other hand, a small contingent of Italian troops (n = 595) in East Timor (1999–2000) experienced ‘only’ 16 dengue cases (probable infections based on serological tests).^39^ Historical records confirm that dengue has affected military units for well over a century: for instance, US forces stationed in Cuba (1904) suffered a dengue outbreak,^51^ as did troops in the Philippines (1907) a few years later.^38^ It should be noted that comparisons across these eras must be made cautiously, as early 20th-century reports often relied solely on clinical observation in the absence of laboratory confirmation, potentially affecting diagnostic specificity.

## Discussion

### Epidemiological history and trends

Dengue continues to pose a significant health threat to military personnel deployed in endemic regions, where the structured health systems and meticulous medical records of armed forces provide a unique advantage for infectious disease surveillance.^52^ This makes military populations a valuable sentinel group for tracking the transmission and evolution of vector-borne diseases like dengue. The risk of dengue is particularly concerning during pre- and post-deployment periods, when undetected infections can compromise troop readiness or facilitate the spread. Across various armed forces, both the prevalence and incidence of dengue infections vary based on geographic location, environmental exposure and preventive practices. For instance, a retrospective study in the French military reported a sharp rise in dengue incidence in 1997 due to outbreaks in French Polynesia and Martinique,^53^ while US military studies have reported an overall incidence of 1.5% during deployments, with seronegative rates prior to deployment helping establish baseline risk.^9^ In one US study, 1.5% of pre-deployment serum samples were seronegative (15 out of 1000), whereas 7.6% seroconverted post-deployment, underscoring the risk of acquisition during overseas missions.^9^ Among US Army Special Operations forces, dengue seropositivity rates rose from 11.2% to 13.2% after deployment, indicating cumulative risk.^54^ Similarly, Indian studies reported a 6-month incidence of 147.4 per 1000 personnel in cantonment settings, with 40% of cases being asymptomatic,^28^ while a cross-sectional study in Puerto Rico identified a seropositivity rate of 66.8%, highlighting high baseline exposure among personnel in endemic zones.^26^

The role of dengue as a significant cause of illness among troops was first recognized during the Spanish-American War in 1898, where US troops experienced widespread febrile illness attributed to dengue.^34^ Dengue continued to disrupt military campaigns throughout the 20th century, most notably during World War II in Asia and the South Pacific, where it led to considerable morbidity and compromised operational readiness.^34^^,^^55^ In the post-war era, major outbreaks were documented among troops stationed in Vietnam, the Philippines, Somalia and Haiti, with reported attack rates reaching up to 80% and convalescence periods extending beyond three weeks in some cases.^7^^,^^14^^,^^34^

As dengue expanded globally in the latter half of the 20th century, its impact on military deployments became increasingly evident, particularly during peacekeeping missions in endemic regions such as Haiti and Somalia during the 1990s.^7^^,^^8^ Despite these historical patterns, gaps remain in the documentation of dengue’s military history. For instance, Sabin’s seminal 1952 research noted dengue occurrences during World War II across India, Panama, the Philippines, Okinawa and Japan.^6^ However, much of this early data originates from conference proceedings or abstract-only records, offering limited methodological detail and hindering inclusion in formal summaries.^56^^,^^57^ These historical gaps highlight the need for improved archival reporting and accessibility of military health data to better understand the evolving burden of dengue in armed forces worldwide.

### Clinical manifestations

The clinical spectrum of dengue in military personnel ranges from asymptomatic infection to severe disease, influenced by prior exposure (i.e. the individuals’ immune status) and environmental stressors (i.e. field conditions). Common symptoms reported in military cases include high fever (up to 100% of symptomatic cases),^58^ intense headache and myalgias in over 90% of patients.^7^^,^^54^ Rash is frequently observed, and gastrointestinal symptoms such as vomiting (35%) and abdominal pain (24%) are also common.^7^^,^^54^ Although most cases are self-limiting, severe manifestations such as DHF and DSS have been observed, especially with repeated deployments to high-transmission regions.^54^ Precise incidence or prevalence data specifically for this group are limited. This is due to underreporting, differences in surveillance systems and varying case definitions across countries. Many cases are also misdiagnosed as other febrile illnesses.^59^ In 2024, WHO reported a significant global burden of dengue, with millions of cases worldwide, including numerous confirmed and severe infections and thousands of associated deaths.^1^ Such severe cases pose significant operational challenges, as they can deteriorate rapidly and often require intensive medical management not readily available in austere field settings. Notably, repeated dengue infections (with different serotypes) are a known risk factor for severe illness; thus, military personnel with multiple deployments in endemic areas warrant careful monitoring for warning signs of DHF/DSS.

### Dengue serotypes

All four dengue virus serotypes (DENV-1 to DENV-4) have been documented in military personnel. In a 1994 outbreak in Haiti, US troops were found to be infected with DENV-1, DENV-2 and DENV-4.^14^ Similarly, in the 1984 Philippines epidemic, multiple serotypes including DENV-1, DENV-2 and DENV-3 were detected in hospitalized service members.^10^ The presence of multiple serotypes in these outbreaks raises the risk of secondary infections for troops who deploy repeatedly to various endemic regions. Seroprevalence studies indicate that up to 9.6% of troops harbour neutralizing antibodies to one or more serotypes, confirming prior exposure (i.e. prior silent or symptomatic infections).^54^ This immunity profile is a double-edged sword: while it may confer some protection, it also means a substantial proportion of personnel are at risk of antibody-dependent enhancement (and thus severe dengue) if re-exposed to a different serotype.

### Diagnostics

Accurate and timely diagnosis of dengue in military context remains a challenge, as diagnostic capabilities vary widely across deployment zones. In resource-limited field environments, laboratory confirmation can be difficult.^12^ While serology (IgM and IgG) remains the most frequently used method, advanced diagnostics including NS1 antigen detection, PCR and viral culture have been adopted in military surveillance systems. French military operations in 2010-2011, for example, employed a full panel of ELISA, PCR, NS1 and cell culture diagnostics across multiple countries.^32^ Hematologic indicators such as thrombocytopenia (up to 92.5%), leukopenia (20.2%) and elevated liver transaminases are commonly seen and may support clinical diagnosis in resource-limited settings.^7^ Symptoms are also observed in other arboviral infections. Notably, most studies applied standard WHO case definitions for dengue when classifying clinical cases. Improvements in portable diagnostics will further enhance field diagnostic capability.^15^

### Treatment and outcomes

There is no specific antiviral treatment for dengue; care is primarily supportive. In military and civilian settings alike, management focuses on fluid replacement and fever reduction, and close monitoring for complications such as haemorrhage or shock.^60^ Early diagnosis and close monitoring are crucial for optimal outcomes, especially in severe cases requiring intensive care.^61^ WHO provides a stepwise approach for managing dengue, emphasizing early recognition of warning signs and aggressive fluid resuscitation and organ support in severe cases.^61^ In deployed military contexts, most cases recover with rest and hydration (often in an outpatient or field infirmary setting). However, severe dengue may require hospitalization, impacting troop strength and readiness. Early diagnosis is thus essential to avoid complications and facilitate timely medical evacuation when needed. In extreme cases with significant bleeding or dangerously low platelets, interventions like blood transfusions may be required, although such resources are limited in the field. Fortunately, in the studies reviewed, fatal outcomes were rare—indicating that with appropriate care, even severe cases can often be managed successfully until recovery or evacuation. However, fatigue experienced during the acute phase of dengue infection may persist as post-infectious fatigue, potentially affecting an individual’s quality of life.^62^ Additionally, each case represents a substantial disruption to operations, underscoring the value of preventive measures to avoid infections in the first place.

### Prevention strategies

Mitigating dengue risk during deployments relies heavily on vector control, PPMs, surveillance and vaccines.

### Vector control

Vector control remains a primary preventive strategy in military environments. Military forces have implemented an array of strategies to reduce mosquito exposure. The French Armed Forces have actively developed advanced techniques for controlling mosquito larvae.^63^ Despite these efforts, widespread insecticide resistance has reduced the efficacy of traditional methods. Although not in a military context, this has prompted interest in novel vector-control approaches such as releasing *Wolbachia*-infected *Aedes* mosquitoes and deploying genetically modified mosquitoes designed to suppress wild vector populations—strategies that are gaining attention for long-term dengue control.^64^^,^^65^ By directly influencing soldier compliance and putting comprehensive disease prevention policies into practice, the chain of command plays a crucial role in vector control. The French military routinely employs this strategy, with command-level mosquito control committees in charge of creating vector control plans specific to regional circumstances.^63^

### Personal protective measures

These include wearing treated clothing and long sleeves, using bed nets and applying topical repellents are essential. The German Armed Forces (Bundeswehr) have likewise updated their standard-issue gear to enhance protection, including insecticide-impregnated uniforms and tent linings.^66^ The French and USA Armed Forces, for example, routinely use permethrin-treated uniforms, DEET-based repellents and insecticide-treated bed nets.^17^^,^^63^^,^^67^^,^^68^ In practice, however, PPM compliance is often inconsistent, especially during intense operations or in challenging tropical environments. This emphasizes the need for continuous education, training and enforcement of preventive medicine protocols in the field to ensure that troops use the protective tools available to them.^8^^,^^67^ According to a study, the use of protective gear, such as DEET and permethrin-treated clothing, by troops is greatly influenced by command emphasis.^69^

### Surveillance

Effective surveillance is another critical component of dengue prevention in military settings. Some armed forces have established dedicated surveillance systems to detect and respond to dengue threats early. For instance, the French military implemented a sentinel surveillance network in West Africa to monitor febrile illnesses among personnel, which successfully identified multiple dengue cases and facilitated prompt vector control responses.^33^ Similarly, syndromic surveillance within the French Armed Forces in French Guiana in 2006 provided early warning of a dengue outbreak, triggering interventions that curtailed its spread.^36^ Active surveillance measures—such as routine pre-deployment and post-deployment serological testing—have been proposed to differentiate between infections acquired abroad versus those locally transmitted after troops return home.^29^ Regular serosurveys and tracking of seroconversion rates in units can offer valuable insights into exposure patterns and outbreak dynamics. Strengthening such surveillance infrastructure in military organizations worldwide would improve situational awareness and allow for data-driven adjustments to preventive strategies during deployments. Dengue is, of course, only one parameter, albeit a very important one. Other differential diagnostic pathogens, such as flaviviruses, are impaired by cross-reactions. Hence, general surveillance for arboviruses is recommended.

### Dengue vaccines

#### Dengvaxia®

After decades of intensive global research, the first dengue vaccine Dengvaxia (CYD-TDV, Sanofi Pasteur), received approval from the US Food and Drug Administration (FDA) on May 1, 2019, marking a significant milestone in dengue prevention efforts.^64^^,^^70^^,^^71^ However, due to Dengvaxia’s risk profile its use had been restricted to individuals with confirmed prior dengue infection and only for individuals 9–16 years old living in endemic areas, with laboratory evidence of past dengue infection.^64^^,^^72^ Approval in the European Union (EU) by the European Medicines Agency was likewise restricted.^73^ Moreover, Sanofi earlier announced the discontinuation of its production of Dengvaxia,^74^ which meanwhile has been implemented because the manufacturer considered the demand to be insufficient for continuing.^75^

Two additional vaccines have since become available (or are nearing approval): Qdenga (TAK-003, developed by Takeda) and Butantan-DV [TV003, developed by the renowned Instituto Butantan in São Paulo, Brazil,^76^^,^^77^ and the US National Institutes of Health (NIH)].^64^

#### Qdenga®

Qdenga (TAK-003, Takeda), an attenuated tetravalent live vaccine based on the DENV-2 backbone, is now approved for use in Argentina, Brazil, the European Union, Indonesia, the United Kingdom, Thailand,^78^ Columbia, Israel, Malaysia, Switzerland and Vietnam,^79^ but not in the United States. Recent real-world data from the multicentric German TravVacNet study have provided the first clinical experience of Qdenga use among travellers, confirming feasibility and good tolerability in a European travel medicine context.^80^ It can be administered regardless of previous exposure and thus offers an important prevention option for dengue-naïve individuals, as it provides broad protection against serotypes DENV-1 and DENV-2^64^^,^^79^ (yet not against DENV-3 and DENV-4; as discussed below). After three years post vaccination, phase III clinical trials of Qdenga demonstrated cumulative efficacy of 62.0% against virologically confirmed dengue (VCD) and 83.6% against hospitalized VCD. During the third year, however, vaccine efficacy declined to 44.7% for VCD while remaining 70.8% for hospitalizations due to VCD.^81^ The WHO position paper on dengue vaccines recommends Qdenga for individuals aged 6–16 years in high-transmission settings.^82^^,^^83^ Prior to travelling to endemic areas, the WHO recommends the vaccine for those with a history of dengue. In contrast, Qdenga can be considered for dengue-naïve individuals aged 4–60, while the WHO advises against vaccinating individuals over the age of 60 until more data is available.^83^ For travellers and military deployments, current perspectives emphasize risk–benefit considerations, particularly for dengue-naïve adults.^84^

Qdenga’s approval marks a major advancement for military medicine: it provides the first opportunity to vaccinate dengue-naïve service members prior to deployment. In late 2022, the vaccine gained European Commission approval (becoming the first broadly indicated dengue vaccine in the EU), and by 2023, German Health Authorities (STIKO- *Ständige Impfkommission*, Standing Vaccination Commission) officially recommended Qdenga for seropositive travellers at risk of dengue exposure—a category that includes military personnel deployed to endemic regions.^85^ With a two-dose series administered subcutaneously three months apart, Qdenga can induce protective immunity against all four serotypes.^83^ Qdenga offers strong protection against DENV-1 and DENV-2 in dengue-naïve individuals but shows limited or no efficacy against the less prevalent DENV-3 and DENV-4 after 54 months of follow-up.^78^ Protection begins 14 days after the first dose and has been shown to last between doses. Therefore, the first dose can be given as little as 14 days before travel to a dengue-endemic area.^83^ Indeed, much like the success seen with pneumococcal and meningococcal vaccination programs in the military,^86^^,^^87^ incorporating dengue vaccination into standard pre-deployment protocols could reduce disease incidence, prevent outbreaks within units, and safeguard mission effectiveness. It will be important, however, to continue monitoring vaccine performance; for instance, post-licensure studies should watch for any serotype-specific risks (one commentary noted a need to monitor for dengue-3 illness in Qdenga-vaccinated seronegative people).^80^ Overall, the advent of an effective tetravalent vaccine that can be given to dengue-naïve individuals is a game-changer for Force Health Protection.^80^ Military organizations should stay abreast of vaccine recommendations and logistics (cold chain, supply, scheduling of doses) to ensure this tool is utilized to its full potential. However, it should be noted that in all likelihood most military personnel deployed to dengue-endemic areas are dengue seronegative. Therefore, since Qdenga’s efficacy in seronegative individuals is significantly lower than in seropositive ones, breakthrough infections must be expected.

### Butantan-DV

Similar to Qdenga, Butantan-DV (TV003) is a live attenuated, tetravalent vaccine. A phase II trial was completed in 2020. This step-wise, randomized, multicenter, double-blind and controlled clinical trial successfully evaluated the vaccine’s safety and immunogenicity.^88^ A phase III trial commenced in 2016. While originally estimated to be completed in November 2024, this randomized, multicenter, double-blind, placebo-controlled study evaluating the efficacy and safety of Butantan-DV^89^ is now in its final stages, and regulatory approval by the Agência Nacional de Vigilância Sanitária, the Brazilian National Health Surveillance Agency, is expected for 2025.^76^ Preliminary data from this ongoing study published in 2024, demonstrated that a single dose of Butantan-DV protects from serotypes DENV-1 and DENV-2 through two years of follow-up, regardless of the dengue serostatus at baseline.^90^ Particularly following a licensing agreement between the Instituto Butantan and the pharmaceutical company Merck Sharp & Dohme, Butantan-DV has gained international recognition. This agreement, *inter alia*, specifies the global regions in which (subsequent to Brazil) the vaccine shall be made available as a priority.^91^

### Other vaccine candidates

Several promising vaccine candidates are still in clinical evaluation, including ChimeriVax Dengue, live-attenuated chimeric constructs like DENV-DENV Chimera (Inviragen), recombinant protein subunit vaccines, and deoxyribonucleic acid–based platforms.^92^ These advances underscore ongoing efforts to broaden prevention against all four dengue virus serotypes. Ensuring the health and readiness of military personnel is paramount, since vector-borne diseases such as dengue can critically undermine operational capacity. Therefore, proactive immunization strategies—particularly the targeted use of Qdenga—offer high-risk groups (e.g. troops deployed to endemic regions) an effective means to reduce both individual cases and unit-wide disease burdens.^85^

### Future directions

Integrating dengue vaccination into routine pre-deployment health preparations is an essential step to protecting military personnel, especially those at high risk. As additional vaccine candidates are refined and new data on efficacy and safety emerge, guidelines should be updated to broaden immunization coverage among troops. In the interim, targeted use of the Qdenga vaccine should be prioritized for troops deployed to dengue-endemic regions (136 countries listed by WHO).^1^

Complementary to vaccination efforts, enhanced dengue surveillance systems must be established or expanded within military health services. This includes implementing both pre- and post-deployment testing protocols for early detection of infections. Regular serosurveys and tracking of seroconversion rates will offer critical insights into exposure patterns and outbreak dynamics within military populations. These can help differentiate between imported and locally acquired infections. Such data-driven approaches will improve situational awareness and allow for timely interventions (e.g. vector control surges or medical evacuation protocols) when dengue is detected among deployed forces.

As climate change reshapes the geographic range and seasonality of *Aedes* mosquito vectors, military operations will increasingly intersect with areas of high dengue transmission. Proactive risk assessments are needed—using climate-based predictive modelling and vector surveillance– to anticipate dengue hazards in future theatres of operation. This can inform deployment strategies and preventive planning (for example, timing missions in seasons of lower mosquito activity or selecting base locations less conducive to mosquito breeding). Additionally, the global research landscape on dengue in military populations remains disproportionately skewed toward US and French studies, with limited data from other nations’ forces and regions. Expanding international collaboration, research participation and transparency, such as during the recent unreported outbreak involving French troops in Burkina Faso,^93^ is crucial for global situational awareness. Finally, continued innovation in field-appropriate diagnostics, including rapid combination antigen–antibody tests aligned with military health system needs, is vital for timely case detection and decision-making in remote or resource-limited settings. Investing in these areas will ensure that militaries are better prepared to face dengue in an era of evolving epidemiology.

### Limitations

This review has several limitations. First, the heterogeneity of the included studies—spanning different eras, regions and methodologies—made direct comparisons challenging. The diagnostic criteria and technologies used to confirm dengue varied considerably, especially between older historical reports and modern studies; early 20^th^-century accounts lacking laboratory confirmation may have misclassified some febrile illnesses as dengue (or vice versa). We attempted to account for such differences qualitatively, but a formal meta-analysis was not feasible due to inconsistent outcome measures. Second, publication bias could be present: dengue outbreaks in military settings might be underreported by some nations or in instances where cases were mild and not investigated, leading to an overrepresentation of severe or noteworthy outbreaks in the literature. Third, most included studies were observational reports without control groups, which limits causal inferences (e.g. about the effectiveness of specific interventions). Finally, data on long-term outcomes (such as chronic health effects or the impact of sequential dengue infections) in military personnel were generally not available. Despite these limitations, by pooling a century’s worth of disparate studies, our review provides a comprehensive overview of dengue’s impact on military forces and highlights critical gaps to address in future research.

## Conclusion

Dengue continues to present significant operational challenges in military settings, with infection risk shaped by deployment geography, environmental exposure, the level of preventive measures in place and limited vaccine coverage. While the US and French Armed Forces dominate the literature on military dengue, more inclusive global (surveillance and reporting) data are urgently needed to fully characterize the threat. A multifaceted prevention strategy combining vaccination, rigorous vector control, personal protection and innovative, quality assured diagnostics (including differential diagnostic relevant pathogens), is critical for mitigating dengue’s impact on troop health and mission success. Future efforts must prioritize robust surveillance, climate-adapted risk assessments and evidence-based preventive policies to ensure force readiness as military operations increasingly venture into dengue-endemic (and emerging) regions. Importantly, many of these interventions will simultaneously protect personnel against other mosquito-borne diseases (such as malaria, yellow fever, chikungunya and Zika), yielding broader benefits for military health and operational capability.

## Supplementary Material

PRISMA_Checklist_(1)_taaf120

## Data Availability

All data generated or analyzed during this study are included in this article.
